# Emerging Therapeutic Strategies Targeting GPX4-Mediated Ferroptosis in Head and Neck Cancer

**DOI:** 10.3390/ijms26136452

**Published:** 2025-07-04

**Authors:** Jaewang Lee, Youngin Seo, Jong-Lyel Roh

**Affiliations:** 1 Department of Otorhinolaryngology-Head and Neck Surgery, CHA Bundang Medical Center, CHA University, Seongnam 13496, Republic of Korea; 2 Logsynk, Seoul 06153, Republic of Korea; 3 College of Medicine, Gyeongsang National University, Jinju 52727, Republic of Korea; 4 Department of Biomedical Science, General Graduate School, CHA University, Pocheon 11160, Republic of Korea

**Keywords:** ferroptosis, GPX4, lipid peroxidation, head and neck cancer, therapy resistance

## Abstract

Ferroptosis, a regulated form of iron-dependent lipid peroxidation-induced cell death, has emerged as a compelling therapeutic strategy to overcome treatment resistance in head and neck cancer (HNC). Glutathione peroxidase 4 (GPX4), a selenoenzyme responsible for detoxifying phospholipid hydroperoxides, plays a central role in blocking ferroptosis and is frequently upregulated in therapy-resistant HNC subtypes. In this review, we examine the multifaceted regulation of GPX4 expression and function, including transcriptional, post-transcriptional, epigenetic, and proteostatic mechanisms. We explore how GPX4 suppression through pharmacologic inhibitors (e.g., RSL3, withaferin A, statins), metabolic stress, or combined therapies (e.g., radiotherapy, EGFR inhibitors, immunotherapy) induces ferroptosis and resensitizes resistant tumors. We also summarize emerging biomarkers, including GPX4, ACSL4, SLC7A11, and NCOA4, that predict ferroptosis sensitivity and may guide patient selection for ferroptosis-targeted therapies. Single-cell and spatial transcriptomics reveal significant intratumoral heterogeneity in ferroptosis susceptibility, underscoring the need for precision approaches. Despite promising preclinical data, challenges such as drug delivery, toxicity, and resistance mechanisms remain. Nevertheless, the ferroptosis-GPX4 axis represents a unique vulnerability in HNC that can be therapeutically exploited. Integrating ferroptosis modulation into personalized oncology may transform outcomes for patients with refractory disease.

## 1. Introduction

Head and neck cancer (HNC) encompasses a group of biologically diverse tumors arising from the mucosal linings of the oral cavity, nasal cavity and paranasal sinus, pharynx, and larynx, and the salivary gland and mesenchymal tissues. It ranks as the sixth most common cancer worldwide, with over 600,000 new cases diagnosed annually and an overall 5-year survival rate of approximately 50% [1,2]. Despite improvements in surgical techniques, radiotherapy, and systemic therapies, including platinum-based chemotherapy and targeted agents like cetuximab, long-term survival outcomes remain suboptimal, particularly for patients with locally advanced, recurrent, or metastatic diseases [3,4]. The limited efficacy of standard therapies is primarily due to intrinsic and acquired resistance mechanisms, including evasion of apoptosis, enhanced DNA repair capacity, and tumor microenvironment-mediated immune suppression [5,6]. Moreover, epithelial-to-mesenchymal transition (EMT), cancer stemness, metabolic plasticity, and increased antioxidant capacity further contribute to therapeutic failure and tumor recurrence [7,8]. Consequently, there is a critical need to explore novel cell death pathways that may be exploited to overcome resistance and improve therapeutic efficacy.

Ferroptosis, first described in 2012, is an iron-dependent form of regulated cell death characterized by the accumulation of lipid peroxides and distinct from apoptosis, necrosis, and autophagy in terms of morphology and molecular features [9,10]. The ferroptotic process is triggered when intracellular iron catalyzes lipid peroxidation through Fenton reactions and is inadequately countered by cellular antioxidant systems, particularly glutathione (GSH)-dependent enzymes [11,12]. Among these, glutathione peroxidase 4 (GPX4) has emerged as the principal ferroptosis suppressor, detoxifying phospholipid hydroperoxides to maintain membrane integrity and redox homeostasis [13,14]. GPX4 is a selenoprotein whose synthesis depends on the availability of selenium and a functional mevalonate pathway that supports selenocysteine-tRNA biosynthesis [15,16,17]. It plays an indispensable role in a wide range of tissues, including neural, hepatic, and epithelial cells, and its deletion results in embryonic lethality in mouse models [18].

Recent evidence indicates that GPX4 is overexpressed in various malignancies, including HNC, and contributes to therapy resistance by buffering oxidative stress, preventing ferroptotic cell death, and sustaining mesenchymal phenotypes [19,20]. Notably, ferroptosis has been implicated in sensitizing resistant cancer cells to conventional treatments [21]. For instance, cisplatin-resistant oral cancer cells show increased dependency on GPX4, and their survival is significantly compromised upon GPX4 inhibition [22]. Moreover, ferroptosis inducers such as RSL3 and ML162, as well as redox-modulating natural compounds like allicin and fucoxanthin, have demonstrated efficacy in reducing HNC cell viability both in vitro and in xenograft models [19,23,24]. Understanding the molecular mechanisms by which GPX4 controls ferroptosis and the factors that regulate GPX4 itself holds significant promise for identifying novel therapeutic vulnerabilities in HNC. This review aims to comprehensively evaluate the functional role of GPX4 in ferroptosis, elucidate the regulatory pathways governing its expression and activity, and explore the translational implications of GPX4-targeted therapy in the management of HNC. We also assess current biomarkers predictive of ferroptotic sensitivity and discuss how integrating ferroptosis modulation with existing therapies could pave the way for personalized oncology approaches in this challenging malignancy.

## 2. Mechanistic Role of GPX4 in Ferroptosis

Ferroptosis is a form of regulated cell death defined by the iron-dependent accumulation of lethal lipid peroxides, primarily in phospholipids containing polyunsaturated fatty acids (PUFAs) [25,26]. Unlike apoptosis, necroptosis, or pyroptosis, ferroptosis is characterized by extensive oxidative membrane damage and is not associated with nuclear fragmentation or caspase activation [10,27]. The distinct biochemical signature of ferroptosis revolves around redox-active iron, lipid peroxidation, and impaired antioxidant defenses—most notably involving GPX4 [28].

**Figure 1:**
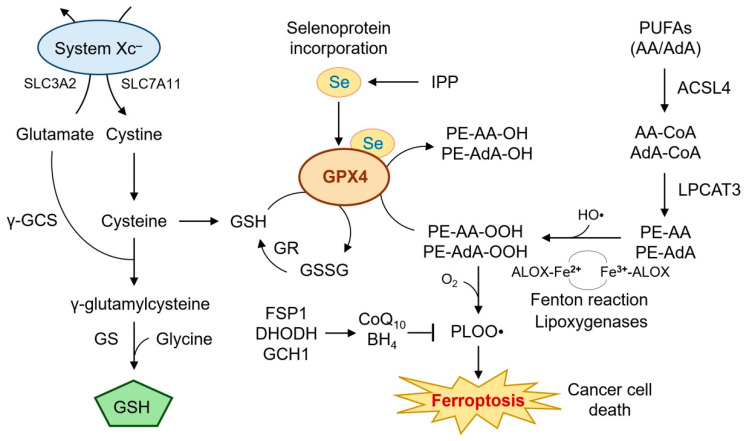
**Mechanistic role of GPX4 in ferroptosis.** This diagram illustrates the central role of GPX4 in suppressing ferroptosis by reducing phospholipid hydroperoxides (e.g., PE-AA-OOH) to non-toxic lipid alcohols. GPX4 activity depends on intracellular GSH, which is synthesized from cysteine provided by the cystine/glutamate antiporter system Xc^−^ (SLC7A11/SLC3A2). The GSH regeneration cycle involving GR and the incorporation of selenium into GPX4 via the mevalonate pathway are also depicted. The availability of GSH is further modulated by glutaminolysis and selenium incorporation into GPX4 via the mevalonate pathway. Lipid peroxidation is driven by iron-mediated Fenton reactions and facilitated by ACSL4 and lipoxygenases. The roles of enzymes such as LPCAT3, ALOX15, and antioxidant defense systems, including FSP1, DHODH, and GCH1/BH_4_, are included to show compensatory pathways under GPX4 inhibition. When GPX4 is impaired, lipid peroxides accumulate, triggering ferroptosis. Additional ferroptosis suppressors such as FSP1, DHODH, and GCH1/BH_4_ provide auxiliary antioxidant defense, especially under GPX4-deficient conditions. This figure integrates metabolic, redox, and lipid regulatory elements central to ferroptosis execution. AA, arachidonic acid; AdA, adrenic acid; ALOX, lipoxygenase; BH_4_, tetrahydrobiopterin; CoQ_10_, coenzyme Q_10_; DHODH dihydroorotate dehydrogenase; FSP1, ferroptosis suppressor protein 1; GCH1, GTP cyclohydrolase 1; γ-GCS, glutamate-cysteine ligase; GPX4, glutathione peroxidase 4; GR, glutathione reductase; GS, glutamine synthetase; GSH, glutathione; GSSG, glutatione disulfide; HO•, hydroxyl radical; IPP, isopentenyl pyrophosphate; LPCAT3, lysophosphatidylcholine acyltransferase 3; PE, phosphatidylethanolamine; PLOOH, phospholipid hydroperoxides; PUFA, polyunsaturated fatty acid; Se, selenium; SLC7A11, solute carrier family 7 member 11; system Xc^−^, cystine/glutamate exchange transporter.

GPX4 is a unique selenoenzyme that catalyzes the reduction of phospholipid hydroperoxides (PLOOHs) to their corresponding alcohols (PLOHs), thereby preventing membrane rupture and ferroptotic cell death (Figure 1) [29,30].

Unlike other glutathione peroxidases, GPX4 can directly detoxify complex lipid peroxides within membranes, a function essential for cell survival under oxidative stress [31]. Knockout models have revealed that GPX4 is indispensable for embryonic development and tissue homeostasis, including neurons, renal tubules, and epithelial linings [18,29]. At the center of this antioxidant defense is the tripeptide glutathione (GSH), which serves as a co-substrate for GPX4. The availability of GSH is tightly regulated by the cystine/glutamate antiporter system Xc^−^, composed of SLC7A11 and SLC3A2, which imports cystine in exchange for glutamate [32,33]. Once inside the cell, cystine is reduced to cysteine and subsequently used for GSH biosynthesis via glutamate-cysteine ligase (GCL) and glutathione synthetase (GSS) [34]. Disruption of this axis impairs GPX4 activity and sensitizes cells to ferroptosis [14,19]. GPX4’s activity is also dependent on selenium incorporation into its active site selenocysteine residue [35,36]. The biosynthesis of selenocysteine-tRNA requires a functional mevalonate pathway, which provides isopentenyl pyrophosphate (IPP) for tRNA modification [37]. Thus, inhibitors of the mevalonate pathway, such as statins, or selenium depletion can attenuate GPX4 function and trigger ferroptosis [38,39].

In addition to its enzymatic detoxification function, GPX4 protects against ferroptosis by limiting the formation of reactive lipid species catalyzed by iron [40]. Cellular iron homeostasis is tightly controlled by the labile iron pool (LIP), transferrin receptor 1 (TFRC), ferritin, and ferroportin (FPN1) [41]. Under ferroptotic conditions, iron-catalyzed Fenton reactions convert hydrogen peroxide into hydroxyl radicals, which initiate lipid peroxidation cascades, particularly in membranes enriched in arachidonic acid (AA) and adrenic acid (AdA) [25,42]. Enzymatic lipid peroxidation is further facilitated by acyl-CoA synthetase long-chain family member 4 (ACSL4), which esterifies AA/AdA into phosphatidylethanolamines (PEs), making them substrates for oxidation by lipoxygenases (ALOX15, ALOX12) [43,44]. In this context, GPX4 functions as the final checkpoint in detoxifying oxidized PEs (PE-AA-OOH), thereby halting ferroptotic execution.

GPX4 expression is notably elevated in resistant tumor phenotypes, including cisplatin-resistant oral squamous cell carcinoma (OSCC) and recurrent nasopharyngeal carcinoma (NPC) [22,45]. Inhibition of GPX4 by RSL3 has been shown to induce ferroptotic death selectively in these cells, with minimal effects on non-malignant cells [19]. This selective vulnerability arises from the heightened oxidative stress and PUFA-enriched membranes characteristic of aggressive tumors [26].

Additional ferroptosis regulators interacting with GPX4 include ferroptosis suppressor protein 1 (FSP1), which regenerates reduced coenzyme Q_10_ (ubiquinol), an alternative lipid ROS scavenger independent of GPX4 [46,47]. Moreover, dihydroorotate dehydrogenase (DHODH) in mitochondria and GCH1-mediated tetrahydrobiopterin (BH_4_) synthesis provide auxiliary antioxidant support, especially under GPX4-deficient conditions [48,49].

Overall, the mechanistic role of GPX4 in ferroptosis reflects a finely tuned balance between antioxidant defenses and pro-oxidant stimuli. Its centrality in maintaining membrane integrity and redox homeostasis positions GPX4 as both a vulnerability and a target in therapy-resistant HNC. The complexity of its regulation—spanning selenoprotein biosynthesis, GSH metabolism, iron homeostasis, and lipid remodeling—offers multiple opportunities for therapeutic intervention, which will be discussed in subsequent sections.

## 3. Regulation of GPX4 Expression and Function in Head and Neck Cancer

**Figure 2:**
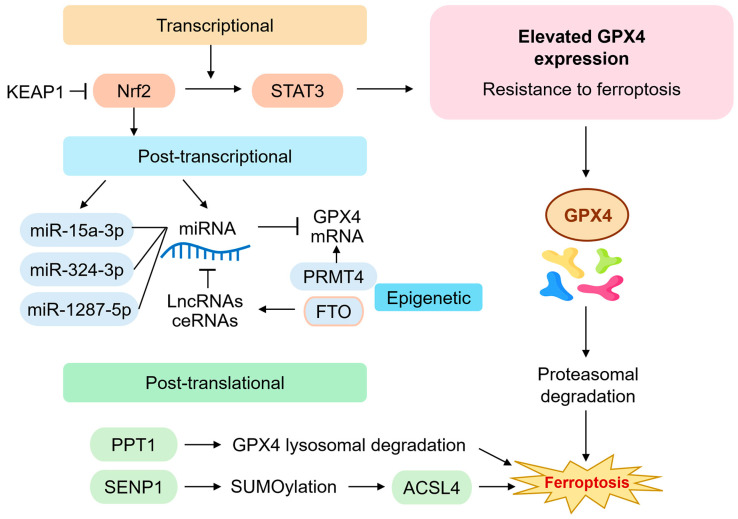
**Regulation of GPX4 expression and function in head and neck cancer.** This diagram summarizes the multilayered regulatory mechanisms governing GPX4 expression in head and neck cancer (HNC). Transcriptionally, GPX4 is induced by Nrf2 binding to antioxidant response elements (AREs) and repressed through KEAP1 or STAT3 inhibition. Post-transcriptionally, non-coding RNAs such as miR-15a-3p, miR-324-3p, and miR-1287-5p downregulate GPX4 mRNA, while lncRNAs (e.g., PVT1, OIP5-AS1) act as molecular sponges that sequester these miRNAs. m^6^A RNA demethylation by FTO and histone modifications by PRMT4 also regulate GPX4 expression. Post-translational control involves PPT1-mediated lysosomal degradation and SUMOylation pathways, with SENP1 indirectly modulating ACSL4 activity and ferroptosis sensitivity. These layers collectively determine ferroptosis resistance in therapy-refractory HNC. ACSL4, acyl-CoA synthetase long-chain family member 4; ceRNA, competing endogenous RNA; FTO, fat mass and obesity-associated protein; Keap1, Kelch-like ECH-associated protein 1; lncRNA, long noncoding RNAs; m^6^A, *N*^6^-methyladenosine; miRNA, microRNA; Nrf2, nuclear erythroid 2-related factor; PPT1, palmitoyl-protein thioesterase 1; PRMT4, protein arginine methyltransferase 4; SENP1, SUMO-specific protease 1; STAT3, signal transducer and activator of transcription 3; SUMO, small ubiquitin-like modifier.

The transcriptional, post-transcriptional, and post-translational regulation of GPX4 is intricately controlled and is critical in modulating ferroptosis sensitivity in HNC (Figure 2). Elevated GPX4 expression has been associated with resistance to conventional chemoradiotherapy and enhanced survival of malignant cells under oxidative stress [6,50,51].

At the transcriptional level, the nuclear factor erythroid 2-related factor 2 (Nrf2) pathway has been identified as a major activator of GPX4 expression. Nrf2 is stabilized under oxidative conditions and translocates to the nucleus, where it binds antioxidant response elements (AREs) in the GPX4 promoter, along with genes encoding SLC7A11 and other antioxidant enzymes [19,52]. Aberrant activation of the Nrf2 pathway in head and neck squamous cell carcinoma (HNSCC) confers resistance to oxidative damage and ferroptosis, particularly in tumors exhibiting mesenchymal features or enhanced EMT [5,53]. Signal transducer and activator of transcription 3 (STAT3) also regulates GPX4 transcription [54]. The inhibition of the PAR1/JAK2/STAT3 axis downregulates GPX4 in HNSCC cancer cells, enhancing their sensitivity to ferroptosis inducers [55,56]. Chromatin immunoprecipitation assays confirmed STAT3’s direct binding to the GPX4 promoter, indicating a transcriptional mechanism relevant to therapeutic resistance [57].

At the post-transcriptional level, non-coding RNAs play a critical regulatory role. Several microRNAs (miRNAs), such as miR-15a-3p, miR-324-3p, and miR-1287-5p, directly bind to the 3′ untranslated region (UTR) of GPX4 mRNA, leading to its degradation or translational repression [58]. Conversely, long non-coding RNAs (lncRNAs), including PVT1 and OIP5-AS1, act as competing endogenous RNAs (ceRNAs) that sponge these miRNAs, thereby stabilizing GPX4 expression [59]. Dysregulation of these RNA networks in HNSCC correlates with tumor progression and resistance to GPX4 inhibitors. m^6^A RNA methylation is another layer of post-transcriptional control. Fat mass and obesity-associated protein (FTO), an m^6^A demethylase, has been reported to increase GPX4 mRNA stability by removing m^6^A marks [60]. The inhibition of FTO reduces GPX4 expression and sensitizes tumor cells to ferroptosis, providing a novel epitranscriptomic mechanism for targeting GPX4 in HNSCC [61]. Epigenetic mechanisms such as histone methylation also influence GPX4 transcription. Protein arginine methyltransferase 4 (PRMT4) enhances GPX4 expression by modifying histone H3 at arginine 17 (H3R17me2a), a mark associated with open chromatin and active transcription [62]. Knockdown of PRMT4 in HNSCC cell lines decreases GPX4 levels and increases sensitivity to ferroptosis-inducing compounds such as RSL3 and erastin [63].

At the protein level, GPX4 is subject to proteasomal and lysosomal degradation. Palmitoyl-protein thioesterase 1 (PPT1) promotes the lysosomal degradation of GPX4, particularly under nutrient-deprived conditions or in tumors with elevated autophagic flux [64]. SUMOylation, a post-translational modification, also regulates GPX4 stability indirectly through effects on ACSL4, a key enzyme involved in PUFA-PE biosynthesis [65]. SENP1, a SUMO-specific protease, stabilizes ACSL4 and promotes lipid peroxidation, thus counterbalancing GPX4’s protective effects [66]. Recent CRISPR-Cas9 screens have identified multiple novel regulators of GPX4 expression and ferroptosis sensitivity, including NCOA4 (ferritinophagy receptor), NRF1 (a regulator of proteasomal genes), and KEAP1 (a repressor of Nrf2) [67]. Single-cell RNA sequencing and spatial transcriptomics analyses further revealed tumor subpopulations within HNSCC that exhibit heterogeneous GPX4 expression, often correlating with immune exclusion and metabolic reprogramming [68].

Clinical transcriptomic data from The Cancer Genome Atlas (TCGA) and other patient-derived datasets support these findings. High GPX4 expression is associated with poor overall survival, radiotherapy resistance, and enrichment of oxidative phosphorylation and lipid metabolism signatures [61,69]. Moreover, GPX4 expression inversely correlates with ACSL4 and NCOA4 levels, suggesting a coordinated regulatory axis influencing ferroptosis outcomes.

Together, these multilayered mechanisms governing GPX4 expression in HNSCC underscore the enzyme’s central role in ferroptosis resistance and highlight potential molecular targets for combination therapy strategies. Future studies should aim to develop predictive models incorporating these regulatory factors for personalized treatment planning.

## 4. Translational and Therapeutic Implications of GPX4 Inhibition in HNC

**Figure 3:**
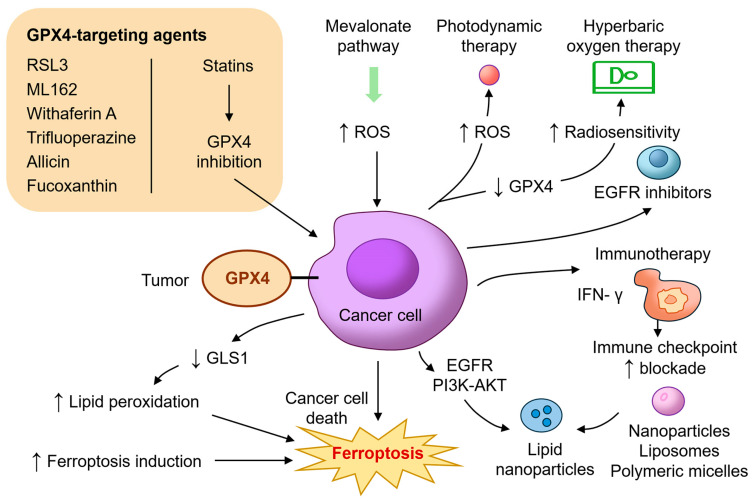
**Translational and therapeutic implications of GPX4 inhibition in head and neck cancer.** This diagram illustrates diverse therapeutic strategies targeting GPX4 and its associated pathways to induce ferroptosis in HNC. GPX4-targeting agents include direct inhibitors such as RSL3, ML162, withaferin A, and trifluoperazine, and indirect modulators like allicin and fucoxanthin that suppress GPX4 expression via the Nrf2/HO-1 axis. Statins inhibit the mevalonate pathway, reducing selenocysteine-tRNA biosynthesis and impairing GPX4 synthesis. Additional mechanisms include glutaminolysis inhibition (e.g., CB-839), photodynamic therapy, and hyperbaric oxygen to promote oxidative stress and radiosensitization. Immunotherapy strategies amplify ferroptosis through IFN-γ-mediated SLC7A11 suppression. Additional combinatorial approaches involving EGFR or PI3K-AKT inhibitors further potentiate ferroptotic responses. Nanoparticle-based delivery platforms enhance tumor selectivity and minimize systemic toxicity. These combined interventions highlight ferroptosis as a promising therapeutic vulnerability in resistant HNC. AKT, protein kinase B; EGFR, epidermal growth factor receptor; GLS1, glutaminase; IFN-γ, interferon gamma; ML162, molecular libraries 162; PI3K, phosphoinositide 3-kinase; ROS, reactive oxygen species; RSL3, RAS-selective lethal 3.

The identification of GPX4 as a critical regulator of ferroptosis has led to intense interest in developing therapeutic strategies that exploit this vulnerability in HNC. Unlike apoptosis, which can be circumvented by common resistance mechanisms such as p53 mutation or BCL2 overexpression, ferroptosis offers a non-redundant pathway of cell death that bypasses traditional evasion strategies [10,14,70]. In this regard, targeting GPX4 or its upstream regulators can sensitize tumors to oxidative damage and trigger ferroptotic death, particularly in refractory HNC subtypes (Figure 3).

Several preclinical studies have demonstrated the efficacy of direct GPX4 inhibitors such as RAS-selective lethal 3 (RSL3) and molecular libraries 162 (ML162) in inducing ferroptosis across a range of cancer models, including HNSCC [71]. RSL3 selectively kills cisplatin-resistant oral squamous carcinoma cells, while sparing normal keratinocytes [22]. Trifluoperazine, a repurposed antipsychotic drug, has also been shown to downregulate GPX4 and trigger ferroptosis through reactive oxygen species (ROS) accumulation and autophagy activation in oral cancer cells [72]. Natural compounds such as allicin and fucoxanthin have emerged as promising agents that inhibit GPX4 via HO-1/Nrf2 pathway modulation, leading to lipid peroxidation and ferroptosis in NPC [23,24]. Withaferin A, another plant-derived compound, binds covalently to the active site of GPX4 and promotes ferroptotic death in resistant cancer types [73,74]. These agents illustrate the potential for both the direct and indirect pharmacologic inhibition of GPX4 in cancer therapy.

In addition to direct inhibitors, targeting the upstream metabolic processes essential for GPX4 synthesis offers another route to ferroptosis induction. GPX4 requires selenocysteine for its enzymatic activity, and this is dependent on the mevalonate pathway and selenoprotein biosynthesis machinery. Statins, which inhibit HMG-CoA reductase and reduce isopentenyl pyrophosphate (IPP), impair selenocysteine-tRNA maturation and suppress GPX4 translation [75,76]. Combination therapies involving statins and ferroptosis inducers have demonstrated synergy in preclinical HNSCC models [69]. Glutaminolysis inhibition represents another complementary approach. Glutaminase (GLS1) inhibition reduces GSH levels, sensitizing tumor cells to ferroptosis. Allevato et al. (2024) demonstrated that CB-839, a GLS1 inhibitor, enhances ferroptosis in HPV-negative HNSCC when combined with GPX4 blockade [68]. Radiotherapy and photodynamic therapy (PDT) both generate intracellular ROS and may synergize with ferroptosis inducers. Wang et al. (2025) showed that PDT using temoporfin induces lipid peroxidation and GPX4 depletion under both normoxic and hypoxic conditions, promoting ferroptosis in HNSCC models [77]. Similarly, Liu et al. (2024) demonstrated that hyperbaric oxygen therapy improves radiosensitivity and induces ferroptosis through the downregulation of GPX4 [78]. Metal ion interference therapy for boosting MRI-guided ferroptosis therapy by inhibiting GPX4 activity has also been developed to allow for real-time monitoring of nanoplatform distribution and accumulation in OSCC [79].

Immunomodulation also intersects with ferroptosis. CD8^+^ T cells promote ferroptosis in tumor cells by secreting interferon-gamma (IFN-γ), which suppresses SLC7A11 and impairs cystine uptake [80]. GPX4 inhibition in this context can enhance immunogenic cell death and potentiate immune checkpoint blockade [81]. This strategy is particularly relevant in HNSCC, where immune suppression and T-cell exclusion are prevalent. Furthermore, several studies have shown that combining GPX4 inhibition with EGFR blockade (e.g., cetuximab) or PI3K-AKT pathway inhibitors results in synergistic ferroptosis and tumor regression [82,83]. Efforts are also underway to develop nanoparticle-based delivery systems to improve the tumor selectivity and bioavailability of ferroptosis inducers. Lipid nanoparticles, liposomes, and polymeric micelles have been engineered to deliver RSL3, erastin, statins, or ginseng specifically to the tumor microenvironment while minimizing systemic toxicity [84,85,86].

From a clinical perspective, the translation of GPX4-targeted therapies into patient care will depend on the availability of reliable biomarkers. Biomarker-driven trials using GPX4, ACSL4, SLC7A11, and other ferroptosis-related signatures may guide patient stratification and treatment response monitoring [69,87]. Clinical trials integrating ferroptosis inducers with standard-of-care treatments in biomarker-selected HNSCC cohorts are warranted. In summary, the therapeutic targeting of GPX4 and its associated pathways presents a promising strategy for overcoming resistance in HNC. A multi-pronged approach combining GPX4 inhibitors, redox modulators, immunotherapies, and smart delivery systems may offer the most robust clinical benefit. As preclinical evidence continues to mount, the integration of ferroptosis into HNC treatment algorithms represents a frontier in precision oncology.

## 5. Biomarkers and Predictive Tools for Ferroptosis Sensitivity in HNC

The application of ferroptosis-inducing therapies in HNC requires a robust biomarker framework to identify responsive patients and monitor therapeutic outcomes. Given the complex, multilayered regulation of ferroptosis, biomarkers must capture genetic, transcriptomic, proteomic, and metabolic dimensions to effectively predict GPX4-related ferroptosis sensitivity.

GPX4 expression itself is a key determinant of ferroptosis resistance. Elevated GPX4 mRNA and protein levels are frequently observed in treatment-resistant HNC, correlating with poor prognosis and radioresistance [50,88]. Transcriptomic data from the TCGA and GEO databases reveal that high GPX4 expression is associated with signatures of EMT, oxidative phosphorylation, and hypoxia—traits commonly linked to therapeutic escape [61,69]. Among these, GPX4 expression itself is a central determinant of ferroptosis resistance. In contrast, the high expression of ACSL4, which facilitates PUFA incorporation into membrane phospholipids, predicts ferroptosis sensitivity [89,90]. ACSL4 acts in concert with lysophosphatidylcholine acyltransferase 3 (LPCAT3) and lipoxygenases to generate oxidized PEs (PE-AA-OOH), the key lipid species implicated in ferroptotic death [91,92]. In HNSCC, tumors with low GPX4 and high ACSL4 show increased response to ferroptosis inducers [66]. Conversely, markers such as ACSL4, SLC7A11, and NCOA4 reflect pathways that either promote or buffer ferroptosis sensitivity.

SLC7A11, a subunit of the cystine/glutamate antiporter system Xc^−^, is another predictive marker. It supplies cystine for GSH biosynthesis, maintaining redox balance and GPX4 activity. High SLC7A11 levels have been correlated with ferroptosis resistance, while its downregulation sensitizes tumors to GPX4 inhibition and radiotherapy [19,93]. NCOA4, a key mediator of ferritinophagy, enhances ferroptosis by mobilizing iron from ferritin stores into the labile iron pool. Low NCOA4 expression impairs iron availability for lipid peroxidation and contributes to ferroptosis resistance [94,95]. TCGA data suggest that NCOA4-low HNSCC tumors exhibit reduced ferroptotic vulnerability, despite high-oxidative-stress signatures [69].

Emerging non-coding RNA biomarkers also provide insight into ferroptotic regulation. For instance, miR-15a and miR-324-3p suppress GPX4 expression by targeting its 3′ untranslated region [58]. LncRNAs such as PVT1 and OIP5-AS1 act as molecular sponges to sequester these miRNAs, stabilizing GPX4 and conferring resistance [96,97]. FTO, an m^6^A RNA demethylase, has been implicated in post-transcriptional GPX4 regulation [98]. High FTO expression is associated with ferroptosis resistance and poor outcomes in HNSCC [61]. In addition to transcript-level indicators, functional protein markers are gaining attention. PPT1 promotes GPX4 degradation via lysosomal pathways, and its upregulation enhances ferroptosis susceptibility [64]. CK19 knockdown in HNSCC cells results in GPX4 downregulation and increased ferroptotic death [88].

Metabolomics and lipidomics markers such as oxidized phospholipids (e.g., PE-AA-OOH) and glutathione redox (GSH/GSSG) ratios in tumor tissues are indicative of ferroptotic priming [99,100]. Additionally, iron assays measuring transferrin saturation, ferritin, and labile iron pool dynamics may offer noninvasive biomarkers of ferroptosis readiness [101,102,103]. These markers offer potential for real-time monitoring in treatment settings.

Furthermore, integrative predictive models such as the ferroptosis-related gene signature (FRGS) and multi-omics classifiers are being developed to stratify patients into high- and low-risk groups. A recent model incorporating GPX4, SLC7A11, ACSL4, FTH1, and NCOA4 stratified HNSCC patients with distinct survival outcomes and therapy responses [69]. Single-cell RNA-seq and spatial transcriptomics have revealed intratumoral ferroptosis heterogeneity, revealing specific subclones with GPX4-driven resistance [68].

Finally, multiplex IHC panels combining GPX4, ACSL4, and SLC7A11 can be applied in clinical biopsies to assess ferroptosis vulnerability [104,105]. These tools represent a translational bridge toward biomarker-guided ferroptosis therapy in HNC. Table 1 summarizes the major biomarkers and their relevance to ferroptosis sensitivity and therapeutic application.

Overall, the development of robust and clinically actionable ferroptosis biomarkers in HNC will be essential for the successful deployment of GPX4-targeted therapies. The continued integration of transcriptomic, epigenetic, and metabolic signatures will enable precision oncology approaches that match ferroptosis inducers with patient-specific vulnerabilities.

## 6. Conclusions and Future Perspectives

HNC remains a formidable clinical challenge due to its high degree of inter- and intratumoral heterogeneity and frequent resistance to standard treatments, such as radiotherapy, chemotherapy, and targeted molecular agents [5]. In recent years, ferroptosis—a form of regulated cell death defined by iron-dependent lipid peroxidation—has emerged as a promising therapeutic modality that circumvents classical resistance pathways, offering new hope for treatment-refractory cancers [20,106]. At the center of ferroptosis regulation is GPX4, a selenoenzyme responsible for neutralizing lipid hydroperoxides and maintaining membrane integrity.

High GPX4 expression has been associated with aggressive tumor features and therapeutic resistance in HNC. The activity and stability of GPX4 are tightly controlled by upstream metabolic processes, including GSH synthesis, selenocysteine incorporation, and iron homeostasis. Multiple regulatory layers, including transcriptional activation (e.g., via NRF2 or STAT3), post-transcriptional mechanisms (e.g., non-coding RNAs), and epigenetic modifications, contribute to GPX4 overexpression and ferroptosis evasion. Preclinical models have demonstrated that both direct GPX4 inhibitors and metabolic or oxidative stress-inducing agents can induce ferroptosis and reduce tumor viability, particularly in mesenchymal or therapy-refractory HNC. Furthermore, combining ferroptosis inducers with radiotherapy, EGFR-targeted drugs, or immunotherapies shows additive or synergistic antitumor effects. The clinical translation of ferroptosis-based strategies will require reliable biomarkers to guide patient selection and treatment optimization. Biomarker candidates include GPX4, ACSL4, SLC7A11, and ferroptosis-related gene signatures, alongside indicators of iron metabolism and lipid peroxidation. Spatial transcriptomic and single-cell analyses may further refine the identification of ferroptosis-sensitive tumor subpopulations.

Several challenges remain before ferroptosis-targeted therapies can be widely adopted. These include optimizing the pharmacokinetics and toxicity profiles of ferroptosis inducers, elucidating the mechanisms of resistance to ferroptotic agents, and integrating biomarker-based selection into clinical trials. Moreover, the real-time monitoring of lipid peroxidation dynamics and ferroptosis biomarkers during therapy will be essential for assessing treatment efficacy. Looking ahead, the convergence of systems biology, CRISPR-based screening, and artificial intelligence-driven modeling is poised to accelerate the discovery of new ferroptosis regulators and refine our understanding of GPX4-related vulnerabilities in HNC and beyond. The integration of ferroptosis modulation into multimodal treatment regimens, especially in patients who are non-responsive to immunotherapy or harbor TP53, KEAP1, or PTEN alterations, represents a strategic opportunity to improve outcomes.

In conclusion, the ferroptosis-GPX4 axis represents both a mechanistic hallmark and therapeutic vulnerability in HNC. By leveraging insights into GPX4 regulation, ferroptosis induction mechanisms, and emerging biomarker platforms, clinicians and researchers may usher in a new era of precision oncology. As we continue to unravel the interplay between iron metabolism, redox biology, and lipid peroxidation, GPX4 stands at the frontier of translational cancer therapeutics, with transformative potential for managing resistant HNC.

Importantly, our findings underscore the feasibility of integrating ferroptosis-targeted approaches into clinical oncology workflows. The ability to pharmacologically or genetically modulate GPX4, especially in tumors refractory to conventional therapies, provides a novel treatment avenue where apoptosis-based regimens fail. Moreover, the identification of robust ferroptosis biomarkers enables rational patient stratification and the design of biomarker-driven clinical trials. This is particularly relevant in HNC subtypes with mesenchymal traits, immune exclusion, or high-oxidative-stress signatures. In this context, ferroptosis-based strategies not only offer therapeutic rescue in resistant cancers but also complement existing modalities such as immunotherapy, radiotherapy, and molecularly targeted agents. Future research should focus on optimizing delivery systems, overcoming resistance to ferroptosis inducers, and integrating ferroptosis vulnerability into next-generation precision medicine platforms.

## Figures and Tables

**Table 1:** Ferroptosis biomarkers and predictive tools in head and neck cancer.

Biomarker/Tool	Biological Role/Function	Ferroptosis Relevance	References
GPX4	Reduces lipid peroxides and maintains redox homeostasis	High expression confers resistance to ferroptosis and poor prognosis	[50,69,85,88]
ACSL4	Facilitates PUFA incorporation into phospholipids, promoting lipid peroxidation	High expression correlates with increased ferroptosis sensitivity	[66,89]
SLC7A11	Transports cystine for GSH synthesis, maintaining antioxidant defense	High expression supports ferroptosis resistance; target for radiosensitization	[19,93]
NCOA4	Mediates ferritinophagy and increases labile iron availability	Loss reduces iron release, decreasing ferroptosis susceptibility	[69,94,95]
miR-15a-3p /miR-324-3p	Targets GPX4 mRNA for degradation, reducing its expression	Suppresses GPX4 and enhances ferroptosis sensitivity	[58]
PVT1/OIP5-AS1	Sponges miRNAs, stabilizing GPX4 mRNA expression	Stabilizes GPX4, promoting ferroptosis resistance	[96,97]
FTO	Removes m^6^A marks to stabilize GPX4 mRNA	High levels are associated with ferroptosis resistance and poor outcome	[61]
PPT1	Promotes lysosomal degradation of GPX4	Enhances ferroptosis via degradation of GPX4 under stress conditions	[64]
CK19	Knockdown leads to GPX4 downregulation and ferroptosis sensitization	Increases sensitivity to ferroptosis inducers upon downregulation	[87]
FRGS model	Integrates ferroptosis-related gene profiles to stratify patient risk	Predicts response to ferroptosis therapy and overall survival outcome	[69]
Glutamine metabolism	Blockade results in the rapid buildup of PUFAs	Increases sensitivity to ferroptosis by GPX4 inhibition	[68]
Oxidized PLs	Indicates ongoing or primed ferroptotic lipid peroxidation	Serves as a direct biomarker of lipid peroxidation in ferroptosis	[99]
GSH/GSSG ratio	Reflects redox imbalance and ferroptotic susceptibility	A decreased ratio indicates ferroptosis priming in tumor cells	[100]
Iron metrics (e.g., ferritin, LIP)	Measures systemic and tumor-associated iron status	Correlates with ferroptosis readiness via iron overload	[101,102]
Multiplex IHC panels	Enables combined biomarker detection in tissue samples	Facilitates clinical profiling of ferroptosis potential	[104,105]

Abbreviations: FRGS, ferroptosis-related gene signature; GSH, glutathione; IHC, immunohistochemistry; LIP, labile iron pool; PLs, phospholipids (e.g., PE-AA-OOH); PUFAs, polyunsaturated fatty acids.

## Data Availability

Not applicable.

## Abbreviations

The following abbreviations are used in this manuscript:

AA	arachidonic acid
ACSL4	acyl-CoA synthetase long-chain family member 4
AdA	adrenic acid
ALOX	lipoxygenases
AREs	antioxidant response elements
circRNAs	circular RNAs; BH_4_, tetrahydrobiopterin
BH_4_	tetrahydrobiopterin
CoQ10	coenzyme Q10 (ubiquinone Q10)
DHODH	dihydroorotate dehydrogenase
EMT	epithelial-mesenchymal transition
FPN1	ferroportin
FRGS	ferroptosis-related gene signatures
FTO	fat mass and obesity-associated protein
GCL	glutamate-cysteine ligase
GLS1	glutaminase
GPX4	glutathione peroxidase 4
GSH	glutathione
GSS	glutathione synthetase
HMGCR	3-hydroxy-3-methylglutaryl-CoA reductase
HNC	head and neck cancer
HNSCC	head and neck squamous cell carcinoma
lncRNAs	long noncoding RNAs
IPP	isopentenyl pyrophosphate
Keap1	Kelch-like ECH-associated protein 1
LIP	labile iron pool
LPCAT3	lysophosphatidylcholine acyltransferase 3
m^6^A, *N*^6^	methyladenosine
miRNAs	microRNAs
mTORC1	mammalian target of rapamycin complex 1
NCOA4	nuclear receptor coactivator 4
ncRNAs	noncoding RNAs
NPC	nasopharyngeal carcinoma
Nrf2	nuclear erythroid 2-related factor
OSCC	oral squamous cell carcinoma
PDT	photodynamic therapy
PEs	phosphatidylethanolamines
PLOOHx	phospholipid hydroperoxides
PLs	phospholipids
PRMT4	protein arginine methyltransferase 4
PPT1	palmitoyl-protein thioesterase 1
PUFA	polyunsaturated fatty acid
ROS	reactive oxygen species
SLC7A11	solute carrier family 7 member 11
STAT3	signal transducer and activator of transcription 3
system xc^−^	cystine/glutamate exchange transporter
TFR1	transferrin receptor 1

## References

[1] Sung H., Ferlay J., Siegel R.L., Laversanne M., Soerjomataram I., Jemal A., Bray F. (2021). Global Cancer Statistics 2020: GLOBOCAN Estimates of Incidence and Mortality Worldwide for 36 Cancers in 185 Countries. CA Cancer J. Clin..

[2] Marur S., Forastiere A.A. (2016). Head and Neck Squamous Cell Carcinoma: Update on Epidemiology, Diagnosis, and Treatment. Mayo Clin. Proc..

[3] Vermorken J.B., Mesia R., Rivera F., Remenar E., Kawecki A., Rottey S., Erfan J., Zabolotnyy D., Kienzer H.R., Cupissol D. (2008). Platinum-based chemotherapy plus cetuximab in head and neck cancer. N. Engl. J. Med..

[4] Kim S.S., Liu H.C., Mell L.K. (2023). Treatment Considerations for Patients with Locoregionally Advanced Head and Neck Cancer with a Contraindication to Cisplatin. Curr. Treat. Options Oncol..

[5] Leemans C.R., Snijders P.J.F., Brakenhoff R.H. (2018). The molecular landscape of head and neck cancer. Nat. Rev. Cancer.

[6] Lee J., Roh J.L. (2022). Induction of ferroptosis in head and neck cancer: A novel bridgehead for fighting cancer resilience. Cancer Lett..

[7] Haddadin L., Sun X. (2025). Stem Cells in Cancer: From Mechanisms to Therapeutic Strategies. Cells.

[8] Wang X., Xue X., Pang M., Yu L., Qian J., Li X., Tian M., Lyu A., Lu C., Liu Y. (2024). Epithelial-mesenchymal plasticity in cancer: Signaling pathways and therapeutic targets. MedComm.

[9] Dixon S.J., Lemberg K.M., Lamprecht M.R., Skouta R., Zaitsev E.M., Gleason C.E., Patel D.N., Bauer A.J., Cantley A.M., Yang W.S. (2012). Ferroptosis: An iron-dependent form of nonapoptotic cell death. Cell.

[10] Stockwell B.R., Friedmann Angeli J.P., Bayir H., Bush A.I., Conrad M., Dixon S.J., Fulda S., Gascón S., Hatzios S.K., Kagan V.E. (2017). Ferroptosis: A Regulated Cell Death Nexus Linking Metabolism, Redox Biology, and Disease. Cell.

[11] Yang W.S., Kim K.J., Gaschler M.M., Patel M., Shchepinov M.S., Stockwell B.R. (2016). Peroxidation of polyunsaturated fatty acids by lipoxygenases drives ferroptosis. Proc. Natl. Acad. Sci. USA.

[12] Stockwell B.R., Jiang X. (2020). The Chemistry and Biology of Ferroptosis. Cell Chem. Biol..

[13] Friedmann Angeli J.P., Conrad M. (2018). Selenium and GPX4, a vital symbiosis. Free Radic. Biol. Med..

[14] Yang W.S., SriRamaratnam R., Welsch M.E., Shimada K., Skouta R., Viswanathan V.S., Cheah J.H., Clemons P.A., Shamji A.F., Clish C.B. (2014). Regulation of ferroptotic cancer cell death by GPX4. Cell.

[15] Shimada K., Skouta R., Kaplan A., Yang W.S., Hayano M., Dixon S.J., Brown L.M., Valenzuela C.A., Wolpaw A.J., Stockwell B.R. (2016). Global survey of cell death mechanisms reveals metabolic regulation of ferroptosis. Nat. Chem. Biol..

[16] Seiler A., Schneider M., Förster H., Roth S., Wirth E.K., Culmsee C., Plesnila N., Kremmer E., Rådmark O., Wurst W. (2008). Glutathione peroxidase 4 senses and translates oxidative stress into 12/15-lipoxygenase dependent- and AIF-mediated cell death. Cell Metab..

[17] Conrad M., Proneth B. (2020). Selenium: Tracing Another Essential Element of Ferroptotic Cell Death. Cell Chem. Biol..

[18] Yoo S.E., Chen L., Na R., Liu Y., Rios C., Van Remmen H., Richardson A., Ran Q. (2012). Gpx4 ablation in adult mice results in a lethal phenotype accompanied by neuronal loss in brain. Free Radic. Biol. Med..

[19] Shin D., Kim E.H., Lee J., Roh J.L. (2018). Nrf2 inhibition reverses resistance to GPX4 inhibitor-induced ferroptosis in head and neck cancer. Free Radic. Biol. Med..

[20] Zhang C., Liu X., Jin S., Chen Y., Guo R. (2022). Ferroptosis in cancer therapy: A novel approach to reversing drug resistance. Mol. Cancer.

[21] Li J., Liu J., Zhou Z., Wu R., Chen X., Yu C., Stockwell B., Kroemer G., Kang R., Tang D. (2023). Tumor-specific GPX4 degradation enhances ferroptosis-initiated antitumor immune response in mouse models of pancreatic cancer. Sci. Transl. Med..

[22] Zhou Q., Wang X., Zhang Y., Wang L., Chen Z. (2022). Inhibition of AEBP1 predisposes cisplatin-resistant oral cancer cells to ferroptosis. BMC Oral Health.

[23] Li X., Luo J.Q., Liao X.Q., Zhang S., Yang L.F., Wu T., Wang L., Xu Q., He B.S., Guo Z. (2024). Allicin inhibits the growth of HONE-1 and HNE1 human nasopharyngeal carcinoma cells by inducing ferroptosis. Neoplasma.

[24] Du H.F., Jiang J.M., Wu S.H., Shi Y.F., Liu H.T., Hua Z.H., Wang C.S., Qian G.Y., Ding H.M. (2024). Fucoxanthin Inhibits the Proliferation and Metastasis of Human Pharyngeal Squamous Cell Carcinoma by Regulating the PI3K/Akt/mTOR Signaling Pathway. Molecules.

[25] Yang W.S., Stockwell B.R. (2016). Ferroptosis: Death by Lipid Peroxidation. Trends Cell Biol..

[26] Lee J., Shin D., Roh J.L. (2023). Lipid metabolism alterations and ferroptosis in cancer: Paving the way for solving cancer resistance. Eur. J. Pharmacol..

[27] Chen F., Kang R., Tang D., Liu J. (2024). Ferroptosis: Principles and significance in health and disease. J. Hematol. Oncol..

[28] Seibt T.M., Proneth B., Conrad M. (2019). Role of GPX4 in ferroptosis and its pharmacological implication. Free Radic. Biol. Med..

[29] Maiorino M., Conrad M., Ursini F. (2018). GPx4, Lipid Peroxidation, and Cell Death: Discoveries, Rediscoveries, and Open Issues. Antioxid. Redox Signal..

[30] Liu Y., Wan Y., Jiang Y., Zhang L., Cheng W. (2023). GPX4: The hub of lipid oxidation, ferroptosis, disease and treatment. Biochim. Biophys. Acta Rev. Cancer.

[31] Nishida Xavier da Silva T., Friedmann Angeli J.P., Ingold I. (2022). GPX4: Old lessons, new features. Biochem. Soc. Trans..

[32] Koppula P., Zhuang L., Gan B. (2021). Cystine transporter SLC7A11/xCT in cancer: Ferroptosis, nutrient dependency, and cancer therapy. Protein Cell.

[33] Liu J., Xia X., Huang P. (2020). xCT: A Critical Molecule That Links Cancer Metabolism to Redox Signaling. Mol. Ther..

[34] Yoon S.J., Combs J.A., Falzone A., Prieto-Farigua N., Caldwell S., Ackerman H.D., Flores E.R., DeNicola G.M. (2023). Comprehensive Metabolic Tracing Reveals the Origin and Catabolism of Cysteine in Mammalian Tissues and Tumors. Cancer Res..

[35] Ito J., Nakamura T., Toyama T., Chen D., Berndt C., Poschmann G., Mourão A.S.D., Doll S., Suzuki M., Zhang W. (2024). PRDX6 dictates ferroptosis sensitivity by directing cellular selenium utilization. Mol. Cell.

[36] Chen Z., Inague A., Kaushal K., Fazeli G., Schilling D., Xavier da Silva T.N., Dos Santos A.F., Cheytan T., Freitas F.P., Yildiz U. (2024). PRDX6 contributes to selenocysteine metabolism and ferroptosis resistance. Mol. Cell.

[37] Warner G.J., Berry M.J., Moustafa M.E., Carlson B.A., Hatfield D.L., Faust J.R. (2000). Inhibition of selenoprotein synthesis by selenocysteine tRNA[Ser]Sec lacking isopentenyladenosine. J. Biol. Chem..

[38] Zheng J., Conrad M. (2020). The Metabolic Underpinnings of Ferroptosis. Cell Metab..

[39] Lee J., Roh J.L. (2024). Cholesterol-ferroptosis nexus: Unveiling novel cancer therapeutic avenues. Cancer Lett..

[40] Zhang W., Liu Y., Liao Y., Zhu C., Zou Z. (2024). GPX4, ferroptosis, and diseases. Biomed. Pharmacother..

[41] Ru Q., Li Y., Chen L., Wu Y., Min J., Wang F. (2024). Iron homeostasis and ferroptosis in human diseases: Mechanisms and therapeutic prospects. Signal Transduct. Target. Ther..

[42] Liang D., Minikes A.M., Jiang X. (2022). Ferroptosis at the intersection of lipid metabolism and cellular signaling. Mol. Cell.

[43] Ding K., Liu C., Li L., Yang M., Jiang N., Luo S., Sun L. (2023). Acyl-CoA synthase ACSL4: An essential target in ferroptosis and fatty acid metabolism. Chin. Med. J..

[44] Wenzel S.E., Tyurina Y.Y., Zhao J., St Croix C.M., Dar H.H., Mao G., Tyurin V.A., Anthonymuthu T.S., Kapralov A.A., Amoscato A.A. (2017). PEBP1 Wardens Ferroptosis by Enabling Lipoxygenase Generation of Lipid Death Signals. Cell.

[45] Chen Y., Feng Y., Lin Y., Zhou X., Wang L., Zhou Y., Lin K., Cai L. (2024). GSTM3 enhances radiosensitivity of nasopharyngeal carcinoma by promoting radiation-induced ferroptosis through USP14/FASN axis and GPX4. Br. J. Cancer.

[46] Bersuker K., Hendricks J.M., Li Z., Magtanong L., Ford B., Tang P.H., Roberts M.A., Tong B., Maimone T.J., Zoncu R. (2019). The CoQ oxidoreductase FSP1 acts parallel to GPX4 to inhibit ferroptosis. Nature.

[47] Doll S., Freitas F.P., Shah R., Aldrovandi M., da Silva M.C., Ingold I., Goya Grocin A., Xavier da Silva T.N., Panzilius E., Scheel C.H. (2019). FSP1 is a glutathione-independent ferroptosis suppressor. Nature.

[48] Kraft V.A.N., Bezjian C.T., Pfeiffer S., Ringelstetter L., Müller C., Zandkarimi F., Merl-Pham J., Bao X., Anastasov N., Kössl J. (2020). GTP Cyclohydrolase 1/Tetrahydrobiopterin Counteract Ferroptosis through Lipid Remodeling. ACS Cent. Sci..

[49] Zhou Y., Tao L., Zhou X., Zuo Z., Gong J., Liu X., Zhou Y., Liu C., Sang N., Liu H. (2021). DHODH and cancer: Promising prospects to be explored. Cancer Metab..

[50] Yuan L., Li S., Chen Q., Xia T., Luo D., Li L., Liu S., Guo S., Liu L., Du C. (2022). EBV infection-induced GPX4 promotes chemoresistance and tumor progression in nasopharyngeal carcinoma. Cell Death Differ..

[51] Wang Y., Hu J., Wu S., Fleishman J.S., Li Y., Xu Y., Zou W., Wang J., Feng Y., Chen J. (2023). Targeting epigenetic and posttranslational modifications regulating ferroptosis for the treatment of diseases. Signal Transduct. Target. Ther..

[52] Dodson M., Castro-Portuguez R., Zhang D.D. (2019). NRF2 plays a critical role in mitigating lipid peroxidation and ferroptosis. Redox Biol..

[53] Lee J., Roh J.L. (2023). Targeting Nrf2 for ferroptosis-based therapy: Implications for overcoming ferroptosis evasion and therapy resistance in cancer. Biochim. Biophys. Acta Mol. Basis Dis..

[54] Xie J., Luo D., Xing P., Ding W. (2025). The Dual Roles of STAT3 in Ferroptosis: Mechanism, Regulation and Therapeutic Potential. J. Inflamm. Res..

[55] Wu K., Liu L., Wu Z., Huang Q., Zhou L., Xie R., Wang M. (2024). Ascorbic acid induces ferroptosis via STAT3/GPX4 signaling in oropharyngeal cancer. Free Radic. Res..

[56] Tian J., Liu C., Li B., Hu N., Gu X., Li D., Ai X., Zhou H., Xiao T., Yang C. (2025). PAR1 inhibition sensitizes HPV-negative HNSCC cells to ferroptosis through inhibition of the STAT3-mediated regulation of iron and lipid metabolic pathways. Oncogene.

[57] Ouyang S., Li H., Lou L., Huang Q., Zhang Z., Mo J., Li M., Lu J., Zhu K., Chu Y. (2022). Inhibition of STAT3-ferroptosis negative regulatory axis suppresses tumor growth and alleviates chemoresistance in gastric cancer. Redox Biol..

[58] Hussain S., Gupta G., Shahwan M., Bansal P., Kaur H., Deorari M., Pant K., Ali H., Singh S.K., Rama Raju Allam V.S. (2024). Non-coding RNA: A key regulator in the Glutathione-GPX4 pathway of ferroptosis. Noncoding RNA Res..

[59] Balihodzic A., Prinz F., Dengler M.A., Calin G.A., Jost P.J., Pichler M. (2022). Non-coding RNAs and ferroptosis: Potential implications for cancer therapy. Cell Death Differ..

[60] Qiao Y., Su M., Zhao H., Liu H., Wang C., Dai X., Liu L., Liu G., Sun H., Sun M. (2024). Targeting FTO induces colorectal cancer ferroptotic cell death by decreasing SLC7A11/GPX4 expression. J. Exp. Clin. Cancer Res..

[61] Wang Z., Li H., Cai H., Liang J., Jiang Y., Song F., Hou C., Hou J. (2023). FTO Sensitizes Oral Squamous Cell Carcinoma to Ferroptosis via Suppressing ACSL3 and GPX4. Int. J. Mol. Sci..

[62] Wang Y., Yan S., Liu X., Deng F., Wang P., Yang L., Hu L., Huang K., He J. (2022). PRMT4 promotes ferroptosis to aggravate doxorubicin-induced cardiomyopathy via inhibition of the Nrf2/GPX4 pathway. Cell Death Differ..

[63] Pu X., Wu H., Liu X., Yang F. (2025). PRMT4 Reduced Erastin-Induced Ferroptosis in Nasopharyngeal Carcinoma Cisplatin-Resistant Cells by Nrf2/GPX4 Pathway. J. Environ. Pathol. Toxicol. Oncol..

[64] Luo Q., Hu S., Tang Y., Yang D., Chen Q. (2024). PPT1 Promotes Growth and Inhibits Ferroptosis of Oral Squamous Cell Carcinoma Cells. Curr. Cancer Drug Targets.

[65] Cui C., Yang F., Li Q. (2022). Post-Translational Modification of GPX4 is a Promising Target for Treating Ferroptosis-Related Diseases. Front. Mol. Biosci..

[66] Xu X., Mao Y., Feng Z., Dai F., Gu T., Zheng J. (2024). SENP1 inhibits ferroptosis and promotes head and neck squamous cell carcinoma by regulating ACSL4 protein stability via SUMO1. Oncol. Rep..

[67] Shakya A., McKee N.W., Dodson M., Chapman E., Zhang D.D. (2023). Anti-Ferroptotic Effects of Nrf2: Beyond the Antioxidant Response. Mol. Cells.

[68] Allevato M.M., Trinh S., Koshizuka K., Nachmanson D., Nguyen T.C., Yokoyama Y., Wu X., Andres A., Wang Z., Watrous J. (2024). A genome-wide CRISPR screen reveals that antagonism of glutamine metabolism sensitizes head and neck squamous cell carcinoma to ferroptotic cell death. Cancer Lett..

[69] Noh J.K., Lee M.K., Lee Y., Bae M., Min S., Kong M., Lee J.W., Kim S.I., Lee Y.C., Ko S.G. (2025). Targeting ferroptosis for improved radiotherapy outcomes in HPV-negative head and neck squamous cell carcinoma. Mol. Oncol..

[70] Lei G., Zhuang L., Gan B. (2022). Targeting ferroptosis as a vulnerability in cancer. Nat. Rev. Cancer.

[71] Roh J.L., Kim E.H., Jang H., Shin D. (2017). Nrf2 inhibition reverses the resistance of cisplatin-resistant head and neck cancer cells to artesunate-induced ferroptosis. Redox Biol..

[72] Tsai S.C., Chang P.C., Lin Y.T., Huang P.T., Chen J.Y., Lin C.S., Wu B.N., Chang H.M., Wu W.J., Chang C.I. (2024). Repurposing of the Antipsychotic Trifluoperazine Induces SLC7A11/GPX4-Mediated Ferroptosis of Oral Cancer via the ROS/Autophagy Pathway. Int. J. Biol. Sci..

[73] Hassannia B., Wiernicki B., Ingold I., Qu F., Van Herck S., Tyurina Y.Y., Bayır H., Abhari B.A., Angeli J.P.F., Choi S.M. (2018). Nano-targeted induction of dual ferroptotic mechanisms eradicates high-risk neuroblastoma. J. Clin. Investig..

[74] Koeberle S.C., Kipp A.P., Stuppner H., Koeberle A. (2023). Ferroptosis-modulating small molecules for targeting drug-resistant cancer: Challenges and opportunities in manipulating redox signaling. Med. Res. Rev..

[75] Jiang W., Hu J.W., He X.R., Jin W.L., He X.Y. (2021). Statins: A repurposed drug to fight cancer. J. Exp. Clin. Cancer Res..

[76] Moustafa M.E., Carlson B.A., El-Saadani M.A., Kryukov G.V., Sun Q.A., Harney J.W., Hill K.E., Combs G.F., Feigenbaum L., Mansur D.B. (2001). Selective inhibition of selenocysteine tRNA maturation and selenoprotein synthesis in transgenic mice expressing isopentenyladenosine-deficient selenocysteine tRNA. Mol. Cell. Biol..

[77] Wang T.H., Chou L.F., Shen Y.W., Lin N.C., Shih Y.H., Shieh T.M. (2025). Mechanistic insights into temoporfin-based photodynamic therapy: Ferroptosis as a critical regulator under normoxic and hypoxic conditions in head and neck cancer. J. Photochem. Photobiol. B.

[78] Liu J., An W., Zhao Q., Liu Z., Jiang Y., Li H., Wang D. (2024). Hyperbaric oxygen enhances X-ray induced ferroptosis in oral squamous cell carcinoma cells. Oral Dis..

[79] Chang Z., Liang Z., Lan Y., Huang J., Feng L., Xu J. (2025). Strategy of “Controllable Ions Interference” for Boosting MRI-Guided Ferroptosis Therapy of Tumors. ACS Appl. Mater. Interfaces.

[80] Liao P., Wang W., Wang W., Kryczek I., Li X., Bian Y., Sell A., Wei S., Grove S., Johnson J.K. (2022). CD8(+) T cells and fatty acids orchestrate tumor ferroptosis and immunity via ACSL4. Cancer Cell.

[81] Zhao Y.Y., Lian J.X., Lan Z., Zou K.L., Wang W.M., Yu G.T. (2023). Ferroptosis promotes anti-tumor immune response by inducing immunogenic exposure in HNSCC. Oral Dis..

[82] Jehl A., Conrad O., Burgy M., Foppolo S., Vauchelles R., Ronzani C., Etienne-Selloum N., Chenard M.P., Danic A., Dourlhes T. (2023). Blocking EREG/GPX4 Sensitizes Head and Neck Cancer to Cetuximab through Ferroptosis Induction. Cells.

[83] Yi J., Zhu J., Wu J., Thompson C.B., Jiang X. (2020). Oncogenic activation of PI3K-AKT-mTOR signaling suppresses ferroptosis via SREBP-mediated lipogenesis. Proc. Natl. Acad. Sci. USA.

[84] Wang D., Tang L., Chen M., Gong Z., Fan C., Qu H., Liu Y., Shi L., Mo Y., Wang Y. (2024). Nanocarriers Targeting Circular RNA ADARB1 Boost Radiosensitivity of Nasopharyngeal Carcinoma through Synergically Promoting Ferroptosis. ACS Nano.

[85] Wang Z., Han J., Guo Z., Wu H., Liu Y., Wang W., Zhang C., Liu J. (2023). Ginseng-based carbon dots inhibit the growth of squamous cancer cells by increasing ferroptosis. Front. Oncol..

[86] Zhang X., Li M., Pang X., Wang W.L., Wang X.C., Shen Z.L., Shi R.J., Tang Y.L., Liang X.H. (2025). An injectable hydrogel with photothermal and chemodynamic therapies for targeted promotion of ferroptosis in oral squamous cell carcinoma. Nanoscale.

[87] Rao Y., Li J., Shi L., Chen X., Hu Y., Mao Y., Zhang X., Liu X. (2024). Silencing CK19 regulates ferroptosis by affecting the expression of GPX4 and ACSL4 in oral squamous cell carcinoma in vivo and in vitro. Sci. Rep..

[88] Chen H., Peng F., Xu J., Wang G., Zhao Y. (2023). Increased expression of GPX4 promotes the tumorigenesis of thyroid cancer by inhibiting ferroptosis and predicts poor clinical outcomes. Aging.

[89] Qiu B., Zandkarimi F., Bezjian C.T., Reznik E., Soni R.K., Gu W., Jiang X., Stockwell B.R. (2024). Phospholipids with two polyunsaturated fatty acyl tails promote ferroptosis. Cell.

[90] Wang Y., Hu M., Cao J., Wang F., Han J.R., Wu T.W., Li L., Yu J., Fan Y., Xie G. (2025). ACSL4 and polyunsaturated lipids support metastatic extravasation and colonization. Cell.

[91] Li D., Li Y. (2020). The interaction between ferroptosis and lipid metabolism in cancer. Signal Transduct. Target. Ther..

[92] Kim J.W., Lee J.Y., Oh M., Lee E.W. (2023). An integrated view of lipid metabolism in ferroptosis revisited via lipidomic analysis. Exp. Mol. Med..

[93] Lee J., Roh J.L. (2022). SLC7A11 as a Gateway of Metabolic Perturbation and Ferroptosis Vulnerability in Cancer. Antioxidants.

[94] Mou Y., Wu J., Zhang Y., Abdihamid O., Duan C., Li B. (2021). Low expression of ferritinophagy-related NCOA4 gene in relation to unfavorable outcome and defective immune cells infiltration in clear cell renal carcinoma. BMC Cancer.

[95] Fang Y., Chen X., Tan Q., Zhou H., Xu J., Gu Q. (2021). Inhibiting Ferroptosis through Disrupting the NCOA4-FTH1 Interaction: A New Mechanism of Action. ACS Cent. Sci..

[96] Li J., Ding S., Li M., Zou B., Chu M., Gu G., Chen C., Liu Y.J., Zheng K., Meng Z. (2024). LncRNA PVT1 promotes malignant progression by regulating the miR-7-5p/CDKL1 axis in oral squamous cell carcinoma. Mol. Cell. Probes.

[97] Hsieh P.L., Chao S.C., Chu P.M., Yu C.C. (2022). Regulation of Ferroptosis by Non-Coding RNAs in Head and Neck Cancers. Int. J. Mol. Sci..

[98] Shi J.X., Zhang Z.C., Yin H.Z., Piao X.J., Liu C.H., Liu Q.J., Zhang J.C., Zhou W.X., Liu F.C., Yang F. (2024). RNA m6A modification in ferroptosis: Implications for advancing tumor immunotherapy. Mol. Cancer.

[99] Alves F., Lane D., Nguyen T.P.M., Bush A.I., Ayton S. (2025). In defence of ferroptosis. Signal Transduct. Target. Ther..

[100] Yang K., Wang X., Song C., He Z., Wang R., Xu Y., Jiang G., Wan Y., Mei J., Mao W. (2023). The role of lipid metabolic reprogramming in tumor microenvironment. Theranostics.

[101] Krijt M., Jirkovska A., Kabickova T., Melenovsky V., Petrak J., Vyoral D. (2018). Detection and quantitation of iron in ferritin, transferrin and labile iron pool (LIP) in cardiomyocytes using (55)Fe and storage phosphorimaging. Biochim. Biophys. Acta Gen. Subj..

[102] Van Kessel A.T.M., Karimi R., Cosa G. (2022). Live-cell imaging reveals impaired detoxification of lipid-derived electrophiles is a hallmark of ferroptosis. Chem. Sci..

[103] Ritho J., Dixon S.J. (2022). Excited to see you: New imaging approaches to detect ferrous iron in vivo. Cell Chem. Biol..

[104] Gupta G., Bhat A.A., Goyal A., Singla N., Gupta S., Sharma S., Bhatt S., Dua K. (2023). Exploring ACSL4/LPCAT3/ALOX15 and SLC7A11/GPX4/NFE2L2 as potential targets in ferroptosis-based cancer therapy. Future Med. Chem..

[105] Banu M.A., Dovas A., Argenziano M.G., Zhao W., Sperring C.P., Cuervo Grajal H., Liu Z., Higgins D.M., Amini M., Pereira B. (2024). A cell state-specific metabolic vulnerability to GPX4-dependent ferroptosis in glioblastoma. EMBO J..

[106] Zhou Q., Meng Y., Li D., Yao L., Le J., Liu Y., Sun Y., Zeng F., Chen X., Deng G. (2024). Ferroptosis in cancer: From molecular mechanisms to therapeutic strategies. Signal Transduct. Target. Ther..

